# Necrotizing Fasciitis Due to Prevotella denticola Infection in an Intravenous Drug User

**DOI:** 10.7759/cureus.20901

**Published:** 2022-01-03

**Authors:** Jeremiah Ling, Takashi Hirase

**Affiliations:** 1 Orthopedics and Sports Medicine, Houston Methodist Hospital, Houston, USA

**Keywords:** abscess, polymicrobial, intravenous drug use, prevotella, necrotizing fasciitis

## Abstract

Intravenous drug users (IVDUs) have an increased risk for various types of local and systemic infections including necrotizing fasciitis (NF), which is a life-threatening bacterial soft tissue infection that requires a prompt diagnosis and treatment. *Prevotella denticola* is a part of the *Prevotella *species, which are obligate anaerobic, Gram-negative rods related to *Bacteroides* genus and often implicated in periodontal and dental disease, but have been associated with soft tissue infections and other systemic complications such as cerebral abscess and endocarditis. This case reports a 30-year-old female IVDU who presented with necrotizing fasciitis of the right anterior thigh with associated right knee septic arthritis due to *Prevotella denticola*. The patient was treated with emergent irrigation and radical debridement along with IV antibiotic treatment for eight weeks. A review of literature was performed regarding necrotizing fasciitis caused by *Prevotella* species. Necrotizing fasciitis caused by *Prevotella *species is rare; however, there must be a high index of suspicion among IVDUs to allow for a prompt diagnosis and treatment.

## Introduction

Intravenous drug users (IVDUs) are at increased risk for skin and soft tissue infections, which are the most common cause of hospital admission within this population [1]. *Prevotella* species are commonly associated with periodontal and oral infections; however, they are also rare causes for skin and soft tissue infections [2]. *Prevotella* are related to the *Bacteroides* genus and are part of a group of anaerobic Gram-negative bacilli that are commonly implicated in human infections [2,3]. Typical infections involving *Prevotella* species are related to periodontal and oral complications, with potential for spread to contiguous regions and related conditions including otitis media and cerebral abscess [2,3]. There have been reports of other complications besides the head and neck region involving *Prevotella* species, including soft tissue infection with *Prevotella loescheii*; however, there have been no previous reports of *Prevotella denticola* causing necrotizing fasciitis (NF).

## Case presentation

This case reports a 30-year-old female IVDU who presents to the emergency department reporting a two-week history of progressively worsening right thigh and knee pain with associated redness and swelling. She reported prior attempts of IV needle injections into the anterior thigh, with the most recent attempt being three days prior to presentation. She denied any fevers, respiratory symptoms, or recent travel. Her vital signs upon presentation were blood pressure of 114/61, heart rate of 149, respiration rate of 16, oral temperature of 100.7°F, and blood oxygen saturation (SpO_2_) of 96% on room air. She was neurovascularly intact on examination with palpable fluctuance and crepitus over her anterior thigh with associated warmth, erythema, and disproportionate pain. A significant knee effusion was also present with severe pain on passive motion of the knee. Laboratory Risk Indicator for Necrotizing Fasciitis (LRINEC) score was 11. 

**Figure 1:**
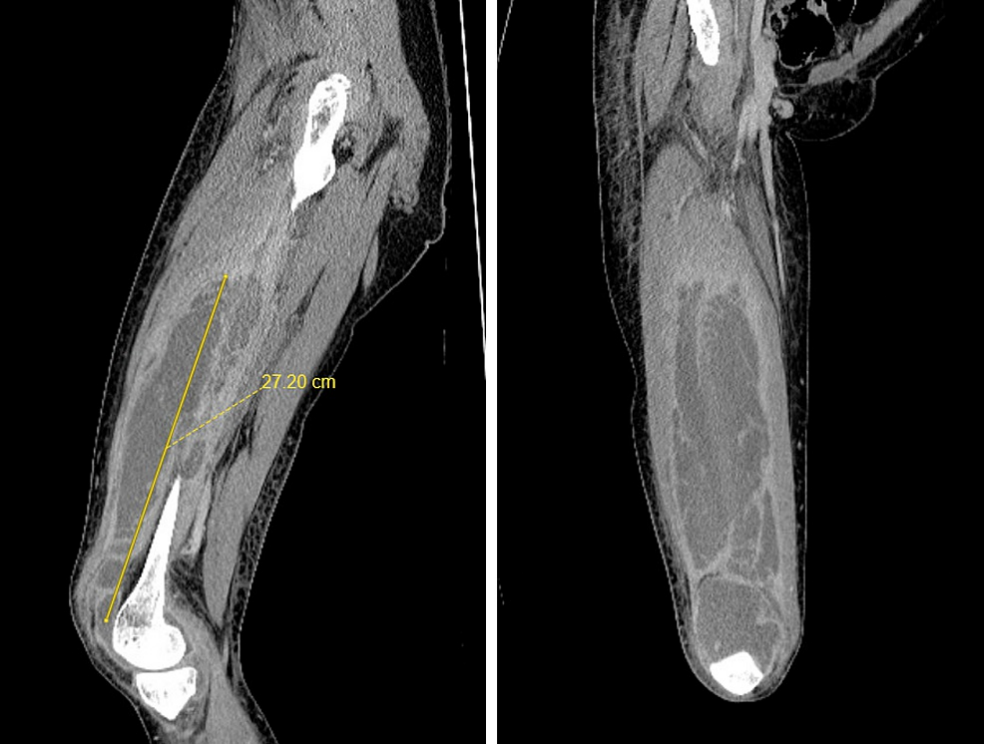
Sagittal and coronal computer tomography cuts of the right femur which demonstrates a large intrafascial abscess within the anterior thigh compartment that extends into the knee joint.

Plain radiographs of the right knee and femur revealed nonspecific soft tissue swelling and knee effusion; however, there was no evidence of soft tissue gas or acute osseous abnormalities. A computer tomography (CT) of the right lower extremity with contrast revealed a large interfascial abscess expanding throughout the anterior compartment of the right thigh with extension into the right knee joint (Figure 1). 

The patient was emergently taken to surgery for irrigation and radical debridement of the right anterior thigh and knee. A midline incision along the right anterior thigh and knee was made and carried out to the anterior compartment. Over 500 mL of dishwater-like purulence was evacuated between the fascial planes of the anterior thigh compartments with associated fascial necrosis. Cultures were sent from the purulence sample. Sharp debridement was performed to excise the necrotic tissue, and the entirety of the right anterior thigh compartment was thoroughly irrigated with 9 L of normal saline. The incision was then carried out distally over the knee to perform an arthrotomy. One hundred milliliters of dishwater-like purulence was further evacuated from the right knee joint with extensive synovitis. Sharp debridement was performed to excise the synovitis, and the entirety of the exposure was reirrigated with another 9 L of normal saline. The wound was closed in a layered fashion using nonbraided sutures, and an incisional wound vacuum (VAC) was placed. She tolerated the procedure well without immediate complications and transferred to the acute care unit with broad-spectrum intravenous (IV) antibiotics consisting of vancomycin, ceftriaxone, and metronidazole. 

Intraoperative anaerobic cultures grew *Prevotella denticola* on postoperative day (POD) 1, and aerobic cultures grew methicillin-resistant *Staphylococcus aureus* (MRSA) and group B streptococcus on POD 2. Acid-fast bacilli (AFB) and fungal cultures did not reveal growth. IV antibiotics were adjusted to vancomycin and metronidazole once cultures were finalized for coverage of all three organisms. A transesophageal echocardiogram was performed on POD 4, which did not reveal masses or vegetations. 

Due to her history of IVDU, discharging the patient on IV antibiotics with a peripherally inserted central catheter (PICC) line was precluded. Thus, she remained inpatient for an additional six weeks to receive IV vancomycin and metronidazole. The incisional VAC was converted to dry dressings at POD 14. At the time of discharge, her surgical incision was well healed without signs of persistent infection. 

## Discussion

To our knowledge, this is the first reported case of NF caused by *Prevotella denticola*. In IVDU, NF remains one of the top reasons for hospitalization and mortality rates range from 9% to 29% [1,4,5]. Severe cases of NF often involve patients with lifestyle choices and other medical comorbidities that put them at risk for more extreme cases; these include obesity, smoking, diabetes mellitus, renal dysfunction, and IVDU [5-7]. Although the microbes involved in NF can vary, there exists a classification system for the more common species involved in NF, which include β-hemolytic streptococci group A and staphylococci species and *Vibrio *and *Aeromonas* species [5,8]. In the case of our patient who was an IVDU, they are at a higher risk for NF; interestingly though, cultures involved in their infection grew *Prevotella denticola *species, which are an obligate anaerobic, Gram-negative rod often found in dental, head, and neck infection and abscess [1,4,5]. A recent case report describes a rarer case of septic emboli related to infection by *Prevotella denticola* following a wisdom tooth extraction [9]. Of note, *Prevotella *spp. have been associated with respiratory samples in cystic fibrosis patients, may be significant contributors to the infection and inflammation of lungs in this patient population, and may be more involved in infection outside of the oral cavity than previously thought [10]. 

In the case of our patient, they denied any recent dental operations and their NF was noted to be confined to their lower extremities. Growth of *Prevotella* spp. was a surprising finding given their lack of recent oral cavity operation; however, given their IVDU status, they are at a higher-risk category for NF. In all patients, NF represents a surgical emergency; in our patient, debridement and excision of necrotic tissues was paramount in early treatment; however, it is important to implement the usage of antimicrobial care in necrotizing fasciitis. In the setting of NF, multidrug antimicrobial therapy usage is paramount and should include agents that cover both Gram-positive and Gram-negative organisms considering that NF is often polymicrobial in nature. In this case report, the authors recommend having a high index of clinical suspicion among IVDU to promptly diagnose and treat polymicrobial NF including those caused by *Prevotella denticola*.

## Conclusions

Necrotizing fasciitis of the lower extremities by *Prevotella denticola* is rare. To our knowledge, this is the first reported case of *Prevotella denticola* infection leading to necrotizing fasciitis in an IVDU. Treatment of necrotizing fasciitis is a surgical emergency, and there must be a high index of suspicion among IVDU to allow for a prompt diagnosis and treatment.

## References

[1] Ebright JR, Pieper B (2002). Skin and soft tissue infections in injection drug users. Infect Dis Clin North Am.

[2] Băncescu G, Dumitriu S, Băncescu A, Preoteasa E, Skaug N (2004). Prevotella species involved in the onset of superficial face and neck abscesses. (Article in Romanian). Bacteriol Virusol Parazitol Epidemiol.

[3] Falagas ME, Siakavellas E (2000). Bacteroides, Prevotella, and Porphyromonas species: a review of antibiotic resistance and therapeutic options. Int J Antimicrob Agents.

[4] Waldron C, Solon JG, O'Gorman J, Humphreys H, Burke JP, McNamara DA (2015). Necrotizing fasciitis: the need for urgent surgical intervention and the impact of intravenous drug use. Surgeon.

[5] Wong CH, Chang HC, Pasupathy S, Khin LW, Tan JL, Low CO (2003). Necrotizing fasciitis: clinical presentation, microbiology, and determinants of mortality. J Bone Joint Surg Am.

[6] Dworkin MS, Westercamp MD, Park L, McIntyre A (2009). The epidemiology of necrotizing fasciitis including factors associated with death and amputation. Epidemiol Infect.

[7] Golger A, Ching S, Goldsmith CH, Pennie RA, Bain JR (2007). Mortality in patients with necrotizing fasciitis. Plast Reconstr Surg.

[8] Lee CY, Kuo LT, Peng KT, Hsu WH, Huang TW, Chou YC (2011). Prognostic factors and monomicrobial necrotizing fasciitis: gram-positive versus gram-negative pathogens. BMC Infect Dis.

[9] Wu JS, Kuo CY, Wu JD (2019). Prevotella denticola septic embolic cerebral infarction after difficult lower wisdom tooth extraction. J Dent Sci.

[10] Sherrard LJ, Graham KA, McGrath SJ (2013). Antibiotic resistance in Prevotella species isolated from patients with cystic fibrosis. J Antimicrob Chemother.

